# Strategies for Maintaining the Coach–Analyst Relationship Within Professional Football Utilizing the COMPASS Model: The Performance Analyst’s Perspective

**DOI:** 10.3389/fpsyg.2019.02064

**Published:** 2019-09-10

**Authors:** Michael Bateman, Gareth Jones

**Affiliations:** School of Sport and Exercise Science, University of Worcester, Worcester, United Kingdom

**Keywords:** sports performance analysis, relationship maintenance, elite sport, soccer, coach, analysts, power relationships

## Abstract

There is a considerable body of research by that has investigated the coach–athlete relationship in sport. However, given the multi-disciplinary nature of modern elite coaching, there is a scarcity of research focusing on the relationship between coaches and other members of the coaching and support team. This study examined the perceptions of six elite professional football analyst’s relationships with their respective coaches. Semi structured interviews utilizing the COMPASS Framework were conducted focusing on Conflict, Openness, Motivation, Preventative Strategies, Assurance, Support, and Social Networks. The results verified that the COMPASS Model of relationship maintenance was applicable to this dyad. Content analysis indicated that there was 215 raw data units comprising of 16 higher order themes across the model which was further broken down into 29 lower order themes. All aspects of the model were found to contribute toward a positively maintained relationship. Having an open relationship underpinned by honesty and being able to provide an opinion was seen as the highest rated attribute that was closely followed by supporting the coach by understanding their requirements for successful coaching practice. Not meeting the coach’s expectations was found to cause conflict and was further highlighted by an inductive analysis that revealed the existence of a relationship that is fundamentally dictated by the coach. Implications of this investigation are that professionals which support elite performers need to set out clear expectations of working practice and hierarchies in order to minimize the chance of internal conflict that can impact on the service levels received by the performer.

## Introduction

O’Donoghue (2010) showcased that performance analysis has become a validated support structure for coaches and athletes and although there is a body of research which investigates the role of the analyst (Bampouras et al., 2012; Wright et al., 2013) and the perceptions of performance analysis within the coaching process (Francis and Jones, 2014; Wright et al., 2016), there is a scarcity of information surrounding the way coaches create, interact and maintain working relationships with performance analysts (Wright et al., 2013). The relationship between the analyst and coach has however, been shown to be so important that coaches would attempt to recruit analysts that they have worked with in previous roles when they gain new employment (Reid et al., 2004; Butterworth and Turner, 2014; Huggan et al., 2015).

Whilst research has identified that elite coaches are supported by a large group of support staff, collaborating data and observations for the benefit of the athletes (Reid et al., 2004; Gustafsson et al., 2008; Bampouras et al., 2012; Clement and Arvinen-Barrow, 2013; Wagstaff et al., 2015), research investigating the relationships present within this group has focused primarily around the coach and athlete (Poczwardowski et al., 2002; Jowett, 2003; Lafrenière et al., 2008; Adie and Jowett, 2010). It has been shown that the emotions, cognitions and behaviors of the persons within this dyad have been shown to be central in developing meaningful relationships (Jowett and Ntoumanis, 2004). Research has also been conducted around the maintenance of relationships, including the conceptualization of the COMPASS Model and Coach–Athlete Relationship Maintenance Questionnaire (CARM-Q) validation process (Rhind and Jowett, 2010, 2012) which has provided valuable insights of coaches within typical dyads in sport.

Jowett and colleagues have employed a number of methodological approaches for the expansive array of research into the coach–athlete relationship. They have mainly centered around the use of questionnaires for model validation purposes (Jowett and Chaundy, 2004; Jowett and Clark-Carter, 2006; Lafrenière et al., 2008; Olympiou et al., 2008; Jowett, 2009; Adie and Jowett, 2010; Jowett et al., 2012b; Rhind and Jowett, 2012; Yang and Jowett, 2013) and interviews for exploratory investigations (Jowett and Meek, 2000; Jowett, 2003; Jowett and Cockerill, 2003; Rhind and Jowett, 2010; Jowett et al., 2012a; Jowett and Carpenter, 2015). All interviews adopted a semi structured format utilizing open ended questions and prompts where required to elicit deeper insight (Patton, 2002). This pattern was followed regardless of the sample size being small (*n* = 2) (Jowett, 2003) or large (*n* = 30) (Jowett and Carpenter, 2015). Of those studies to undertake interviews, the selection criteria for inclusion ranged from a minimum 6-month coach–athlete relationship (Jowett et al., 2012a; Jowett and Carpenter, 2015) to 4 years (Jowett and Cockerill, 2003). Jowett and Cockerill’s (2003) research only interviewed athletes. This enabled free expression of the athletes, without worry of their coaches’ involvement or retort (McKenna and Mutrie, 2003). Rhind and Jowett (2010) recruited unconnected coaches and athletes as previous studies had shown this amplified the range and scope of the data to make it more generalizable in the wider sporting context.

Whilst most interviews were conducted in person, Jowett et al. (2012a) utilized telephone interviews for data collection as the data had not been shown to produce significantly different data to interviews in person (Sweet, 2002). The authors did concede that the format may have limited the expression of experiences and impacted on the perception of the information. Content analysis (Weber, 1990) was used extensively (Jowett and Meek, 2000; Jowett, 2003; Jowett and Cockerill, 2003; Rhind and Jowett, 2010; Jowett et al., 2012a) to categorize the data points into a hierarchy of responses and determine a frequency analysis and is well documented as a common approach for sports psychology studies (Côté et al., 1993). This type of processing was found to highlight prominent themes across responses from all participants (Rhind and Jowett, 2010).

Jowett and Meek (2000) investigated the constructs of closeness, co-orientation and complementarity (3C’s) when interviewing four atypical dyads (married couples who also held a coach–athlete relationship) and identified that the coach–athlete relationship was interdependent. Closeness referred to the emotions of the dyad and the affective elements of the coach–athlete relationship were reflected in mutual feelings of trust and respect. Co-orientation was reflective of the quality of the coach–athlete relationship that was highlighted through the thoughts and perspectives of the dyad where mutual empathetic feelings suggested a more effective relationship. Complementarity considered the interactions and co-operative behaviors of the coach and athlete. Where corresponding behaviors were elicited, an increase in the strength of the relationship was perceived. Interdependence is characterized in the coach–athlete dyad by the high levels of closeness, co-orientation and complementarity creating a mutually beneficial relationship at an affective, cognitive and behavioral level (Jowett et al., 2012a). Although the qualitative method provided a rich source of information, useful in advancing the scope of knowledge in the area, including the web of interactions that crossed the 3C’s, the small sample size and use of atypical participants meant that there was a need for a more quantitative study using greater sample size to create generalizable results (Jowett and Meek, 2000). Further validation of the interactive aspects of the 3C’s was explored via interviews with 12 elite Olympic medalists where connections between the C’s were uncovered, although the causality could not be confirmed (Jowett and Cockerill, 2003). They did establish that the relationships contained attributes including, but not limited to, respect, trust, and clear roles. Inconsistencies in the data collection method (some interviews and some written responses to questions) may have impacted upon the validity of the results. The findings were however, suggested to be integral in the initial construct of the Coach–Athlete Relationship Questionnaire (CART-Q) (Jowett and Ntoumanis, 2004) where co-orientation (+1C) was validated as commitment and that has been used as the basis for various empirical research (Jowett, 2009; Rhind and Jowett, 2010; Yang and Jowett, 2013). The conceptualization of the 3+1C’s model (Jowett, 2007) to include co-orientation which measured how accurately the coach/athlete was able to understand how the other was feeling, thinking and behaving. This led to the requirement for better understanding of how these relationships use strategies including conflict management and socializing to keep a dyad in a specific way (Dindia and Canary, 1993).

Keeping a relationship in a stable state using effective maintenance strategies, facilitated long term, satisfying relationships (Canary and Stafford, 1994, p. 4). Rhind and Jowett (2010) investigated how coaches and athletes used such approaches to preserve a steady coach–athlete environment. The authors based the categorization of themes for their research on previous work into romantic relationship maintenance (Stafford and Canary, 1991; Canary and Stafford, 1994) and led to the conceptualization of the COMPASS model. The original categories were: Conflict management (proactive and reactive), openness (discussing feelings), motivation (effort, motivate the other, fun and demonstrations), positivity (adaptability, fairness and external pressure), advice (sport focused, reward feedback and constructive criticism), support (assurance, Sport specific, personal), and social networks (socializing and shared networks).

The “COMPASS” model complemented the 3+1C’s model and was proposed as a method to understand and manage the complex dynamics of the coach–athlete dyad. The authors also identified the potential for the COMPASS model to be transferable to other interpersonal sporting relationships (Rhind and Jowett, 2010). This body of work was further validated by Rhind and Jowett (2012) with the development of the CARM-Q. The authors found some inconsistencies of the COMPASS model after an exploratory factor analysis had identified highly related subscales (Stafford et al., 2000). Positivity was replaced by Preventative Strategies as positivity was found across various subscales of other categories and Advice was replaced by Assurance to differentiate between support and openness which was validated by Study 2 in the paper.

Research within sport has investigated the domineering power exerted by coaches over their athletes (Johns and Johns, 2000; Cushion and Jones, 2006; Rylander, 2016). A number of theories have contributed to a large body of research on power within social psychology that have often been influenced by the seminal work of French and Raven (1959). Their 5 stage model of interpersonal influence depicted the bases of power which were identified as expert, referent, legitimate, coercive and reward. Rylander (2016) interpreted these for research within the coach–athlete setting and defined expert power as the coaches knowledge which the athlete requires or values. Referent power was based around the coach being identified as a role model or somebody that they could relate to creating reciprocal behavioral and actions. Legitimate power was shown as the recognition within society that the athlete was expected to be comply with their direction. Coercive and reward power were seen as the ability of the coach to discipline or reward athlete behavior based on conformity or outcome. This model has undergone several amendments (Raven, 1965, 1992, 1999) over time and have come under some scrutiny (Patchen, 1974; Carson et al., 1993; Yukl, 2006) although the original model is still considered to be applicable for contemporary research (Groom et al., 2012). Cushion and Jones (2006) found that players within a professional football academy were hegemonized by the historical culture which had existed within the industry for years. The social control elicited by a coach has been further investigated (Purdy et al., 2008; Purdy and Jones, 2011) in adult sport and is perceived to be more transient with the idea of power being negotiable between the coach and athlete depending on the circumstance (Poczwardowski et al., 2002; Cushion et al., 2003; Purdy et al., 2008). Jones (2006) also argued that the coach perceived that they were required to interact socially with other contextual stakeholders such as technical staff to maintain their status of power. This is in accordance with Giddens (1984) conceptualization of power that showed the subservient parties had the ability to change the balance of power relationships through social interactions. This was further validated by Purdy et al. (2008) when the athletes showed resistance in addition to compliance and co-operation to a coach in turmoil (Locke, 1985).

The concept of the power dynamic struggles between asymmetrical relationships is well documented and as a result of hierarchical relationships (Cushion et al., 2003; Jones et al., 2009). However, it was suggested that a strong relationship identified through the 3+1C’s model (Jowett, 2007) could negate the conflict that has often been associated with this type of dyad which would result in the coach often looking to teach and guide the protégé (Jowett et al., 2012a). McCalla and Fitzpatrick (2016) argued that although micro-politics are prevalent within high performance teams, when a synergy exists between the value of each contributing facet, and the experts of each field work within their remit, the potential of the staff can contribute to harmonious working environments. However, if the boundaries become blurred, then anxiety and confusion could develop (Frost et al., 2005).

Wright et al. (2013) examined 48 completed questionnaires from performance analysts and found that 89.4% of participants stated that the relationship between the coach and analyst was “Very Important.” They discovered that analysts were consulted to varying degrees within professional football but very rarely led feedback sessions. They summarized that this was due to a number of factors including coaches trust in the analyst, but suggested further investigation was required. They also spoke of the negotiations around the design of analytical processes and defining measures of successful performance. This collaborative workflow contradict the results of Bampouras et al. (2012). Whilst they acknowledged the relationship was instrumental for analytical system success, the perception was that the analyst was responsible for purely collecting the information as directed by the coach. This idea of an unbalanced power relationship has been further supported by Huggan et al. (2015) when an analyst considered the value of the role and their own self-esteem due to the dominance of the coach in the applied setting.

Sarmento et al. (2015) provided evidence of the numerous benefits and attributes that analysts brought to a technical team. However, of the six coaches interviewed, only one coach had access to a full-time analyst which suggests other coaches may have based this opinion on beliefs or past experiences, rather than current circumstance. Huggan et al. (2015) had first hand narrative evidence from a Performance Analyst which reported the participant placed an emphasis on creating strong working relationships with cooperative and supportive colleagues. Manzanares et al. (2014) concluded that bridging the gap between the scientific analysis and the practical coaching process would be mutually beneficial and would only serve to enhance the development of athletes performance. The authors acknowledged the workload and resources required by the analysts within elite sport that was required to support the needs of the coach to make reliable technical and tactical appraisals of performance. Fernandez-Echeverria et al. (2017) have criticized the lack of studies which consider how the analyst is utilized by coaches within the coaching process. These suggestions have been highlighted in football, particularly as the role of a dedicated performance analyst is now a necessity in the current staffing structure (Reeves and Roberts, 2013).

As performance analysts have become more integral to the coaching and feedback process, Hughes (2004) indicated that analysis has interjected into the coach–athlete relationship. They found that the addition of another stakeholder in the process (gathering and analyzing data) allowed greater information share which is instrumental in developing teams. For this to be a successful working relationship, mutual trust and respect must be observed for all parties (Francis and Jones, 2014).

The research outlined above has provided evidence that the quality of the relationships coaches have with their athletes, and their ability to maintain them at a good level, has a correlation with the success that the athletes are able to attain. This coupled with the literature that identifies the performance analyst to be a key component in the coaching process justifies the need to examine the relationship of the coach and the performance analyst to investigate if maintenance strategies are transferable across to this dyad. There is limited research into the relationships between coaches and members of their sports science support staff, so this paper will aim to fill a gap within the literature that can further enhance the coaching process and expand the knowledge base within performance analysis and coaching. The aim of the study is to investigate the relationship between the coach and performance analyst within professional football. Specifically, the research is looking to confirm behaviors that relate to maintenance strategies within this dyad.

Aim 1. Investigate the main components of maintaining the coach–analyst relationship.

Aim 2. Determine if the conceptualized coach–athlete COMPASS model is adaptable to the coach–analyst relationship.

Aim 3. Understand any other pertinent trends which arise from the data.

## Materials and Methods

### Participants

Expert purposive sampling was employed to enlist six current first team performance analysts working full time in professional football clubs in England. By stipulating first team analysts, the researcher was able to gain understanding from participants with significant experiences which hoped to enrich the data provided thorough insight into coach analyst relationships at the highest levels of professional sport (Simonton, 1999; Jowett and Cockerill, 2003). They were also required to have maintained a current or past coach analyst relationship for a minimum of 6 months (Jowett et al., 2012a, b; Jowett and Carpenter, 2015). The selected contributors were recruited personally due to pre-existing relationships with the researcher who had a previous career within the industry. Participants received an information pack which clarified the aims of the study, included an information sheet about the underlying 3+1C’s model and an informed consent sheet.

The six participants ranged in age from 25 to 37 years with a mean age of 30.3 years (±4.88 years) and had an average of 8.16 years (±3.2 years) experience working as a performance analyst within professional football. Five out of the six interviewees had worked for four professional teams and the sixth had worked for two teams during their nine years’ experience. The participants current coach analyst relationships ranged between 3 and 24 months (mean = 10.3 ± 7.5 months). The shortest dyadic working relationship encountered during the analyst’s careers was acknowledged as 21 days by A3 who was also involved in the longest continuous relationship (5 years). The average for the shortest relationship was 84 days (±46 days) and 30 months (±17.69) for the longest relationship.

### Procedures

Ethical approval was granted by the University of Worcester’s ethics and research governance committee to undertake this study. Written and voluntary informed consent was obtained from all participants within the study. The interview questions were validated through a pilot interview with an experienced performance analyst (greater than 10 years’ experience) to assist in identifying any epistemological concerns (Kim, 2010). This initial authentication process resulted in the addition of one question to the schedule to improve the balance of the questions being asked.

The interviews were conducted via telephone (O’Donoghue, 2010, p. 39; Jowett et al., 2012a). This style of data collection negated any restrictions on participation based on the analyst location and has been validated as useful for shorter, focused interviews with busy people (Miller, 1995). The duration of the interviews lasted between 21 min, 14 s and 50 min, 25 s. Semi structured, open ended questions were used with prompts where required based on the conceptualized COMPASS model, the validation of the categories via the CARM-Q framework (Rhind and Jowett, 2010, 2012) and the 3+1C’s framework (Jowett, 2007). The interview was split into three parts; an introductory section to build rapport with the interviewees and collect demographic information (Kvale and Brinkmann, 2009, pp. 123–125). Secondly, the main section of the interview asked four questions pertaining to the analyst’s perceptions and experiences of relationship maintenance strategies (Supplementary Data Sheet 1). Finally, the participants were offered an opportunity to provide any further information they deemed pertinent to the investigation.

An independent coder who had experience of both the applied Performance Analyst role (3 years) and theoretical Academic role (2 years) was employed to assess the raw data units obtained from interview one to ensure the results were not biased via the researcher’s knowledge or experiences (Woods and Thatcher, 2009; Jones et al., 2014). Fifty-seven deductive data units and forty inductive data units were investigated and categorized by the coder which resulted the re-categorization of two units.

Owing to the researchers 10 years’ experience of working within professional football, knowledge of all participants annulled the need to spend time *in situ* building rapport with the analysts. This closeness and prior knowledge of the industry could have led to personal bias, so the researcher practiced bracketing of their opinion through the pilot study and continued this during the main study (Tufford and Newman, 2010). Transcripts of all interviews were sent to the respective interviewee to confirm the accuracy of their account. All participants had their data anonymized whereby each interviewee was referred to as A1–A6 and where names were referred to, pseudonyms were used where required to hide the true identity of those involved.

### Data Analysis

Content analysis was used to systematically discover themes within the data (Weber, 1990, p. 41). After reviewing the interview transcriptions, a deductive content analysis identified themes and sub themes based upon previous research (Rhind and Jowett, 2010, 2012). An inductive approach was also used to identify themes previously not recorded (Rubin and Rubin, 1995; Elo and Kyngäs, 2008). 215 meaningful units were found in the deductive analysis and 142 units were created through the inductive analysis. All units were highlighted and catergorised accordingly.

## Results and Discussion

### “COMPASS Model” in the Coach–Analyst Relationship

The coded results showed (Table 1) that raw data units were divulged for all seven themes within the amended “COMPASS Model” (Rhind and Jowett, 2010, 2012). All six analysts provided insights regarding six of the seven themes yielding varying levels of raw data units ranging between 6 and 18.1% of the total raw units. In percentage contribution order, the results were; Support (18.1%), Preventative Strategies (16.3%), Motivation and Social Networks (14.5%), Conflict Management (11.2%), and Assurance (6%). of the total raw data, Openness provided statements by five out of six participants although it had the highest total number of raw data units of any of the themes commanding 19.5% of the total number of units.

**TABLE 1:** Summary of the results from the COMPASS Model.

**Frequencies**				**Frequencies**				**Frequencies**			
**Compass model**	***n***	**%**	**Analysts**	**Higher order**	***n***	**%**	**Analysts**	**Lower order**	***n***	**%**	**Analysts**
Conflict management	24	11.2	6/6	Reactive	24	11.2	6/6	Consequences of unmet expectations	9	4.2	4/6
								Diffusion of conflict	15	7.0	5/6
Openness	42	19.5	5/6	Non-sport communication	19	8.8	5/6	Honesty	14	6.5	5/6
								Trust	5	2.3	3/6
				Talk about anything	23	10.7	5/6	Approachability	13	6.0	3/6
								Providing your opinion	10	4.7	5/6
Motivation	31	14.4	6/6	Effort	7	3.3	3/6	Commitment to the coach	6	2.8	3/6
								Positivity	1	0.5	1/6
				Fun	4	1.9	2/6	Humor	4	1.9	2/6
				Motivate the other	9	4.2	5/6	Togetherness	6	2.8	3/6
								Multidisciplinary team togetherness	3	1.4	2/6
				Show ability	11	5.1	4/6	Hard work	11	5.1	4/6
Preventative strategies	35	16.3	6/6	Avoiding conflict	14	6.5	6/6	Awareness of coach expectations	5	2.3	3/6
								Submissive avoidance	9	4.2	4/6
				Relationship continuity	8	3.7	5/6	Establishing early understanding	4	1.9	4/6
								Judging dyadic preferences	4	1.9	3/6
				Respecting boundaries	13	6.0	4/6	Respecting others	11	5.1	4/6
								Solution based opinion	2	0.9	1/6
Assurance	13	6.0	6/6	Constructive feedback	1	0.5	1/6	Advice	1	0.5	1/1
				Sport communication	12	5.6	6/6	Relationship building phase	4	1.9	3/6
								Providing analytical expertise	8	3.7	5/6
Support	39	18.1	6/6	Person support	10	4.7	3/6	Mutual respect	4	1.9	2/6
								Unconditional support	6	2.8	2/6
				Sport support	29	13.5	6/6	Communication	5	2.3	4/6
								Understanding coach requirements	24	11.2	5/6
Social networks	31	14.4	6/6	Socialization	4	1.9	1/6	Maintaining previous relationships	4	1.9	1/6
				Shared network	27	12.6	6/6	Casual interactions	10	4.7	5/6
								Obligatory socialization	7	3.3	3/6
								Socialization through sport	10	4.7	4/6
Total	215	100.0		Total	215	100.0		Total	215	100.0	
											

#### Conflict Management

Conflict management was responsible for the reactive elements of maintaining relationships. This is in contrast to Rhind and Jowett (2010) as the pro-active statements were later considered to be their own category (Rhind and Jowett, 2012). It correlated the findings of the subcategory “Consequences of Unmet Expectations” with nine observations (4.2%). These ranged from not allowing conflict to affect the relationship by A1 who stated:

“*if I overstepped the mark, he would either let it go, or he would pull you up on it but it wouldn’t ever be a negative thing*”

Through to severe consequences that ended the association for A3:

“*When you stop being part of a group, or the group I suppose, then I found myself out the door with my p45 so you know, there’s cause and effect*”

Five of the six interviewees reported attempts to diffuse conflicts once they had arisen to enable the relationship to be maintained. These predominately focused around confronting the issue:

“*if you do have an issue, that it’s not just kind of brushed under the carpet, it’s raised in the right way, communicated in the right way and its dealt with at the appropriate time*” (A6: 8 of 15 data units in this sub category)

Or trying to provide solutions with various statements beginning in this manner:

“*Now I tried to resolve this*…” (A3)

“*I think there was smaller things in place where I’d try and get round it*…” (A2)

“*I ended up realising that all I had to do*…” (A5)

The attempts to resolve the situations through co-operative acts was in correlation with the findings of Rhind and Jowett (2010). This was also confirmed by Jowett (2003) who found that if either member of the dyad was unwilling to try and understand the source of the conflict, then the relationship could not be restored to its previously successful state. This studies results suggest the analysts were able to engage in these conversations although the threat of being dismissed due to conflict was still evident from two analysts.

#### Openness

Openness contained two of the three sub categories that were also reported by Rhind and Jowett (2010). Other awareness (understanding how the coach is feeling) was not conveyed although 42 units highlighting both non-sport communication (8.8%) and talk about anything (10.7%) themes were present. The two main subcategories to emerge from non-sport communicating were honesty and trust which centered around discussion of topics that were still work related (Rhind and Jowett, 2010). Examples were provided by A4, A5, and A6 respectively:

“*have that honesty and openness between the both of you to try and improve what you’re doing*”

“*But in terms of having conversations that I know, they wouldn’t want to leave them four walls, you’ve obviously been in plenty of them, and I guess that’s a good sign, that you’re trusted and accepted*”

“*I think the key things trust between the two, the coach and the analyst. So, they’ve got to be able to have two way trust in each other so the coach can trust that the analyst is giving them good information and the analyst trusts that the coach is going to use it and use it the correct way*”

For the analyst, the thought of being trusted and respected was paramount in maintaining open relationships with the coach. This perception of closeness (Jowett and Meek, 2000) was imperative for the analyst to feel they were deemed competent by the coach (Jowett and Cockerill, 2003) and has been shown to differentiate between harmonious and non-harmonious relationships (Canary and Stafford, 1994; Francis and Jones, 2014). These findings built upon Wright et al.’s (2013) call for more research into the trust between the coach and analyst. The present results may have been found due to the length of the dyadic relationships which were not reported within the Wright study.

“Approachability” and “Providing Your Opinion” materialized within the “Talk About Anything” sub category although the overriding premise still surrounded work-based conversations. A6 talked extensively (6 of 13 units) about approachability and examples included:

“*if you’ve got a good relationship with someone, regardless of if you agree with them. I mean, it can go both ways. You can get on with someone personally but not agree with them professionally, or the other way around, but as long as you’ve got at least one of those relationships, you can be, you can have that conversation*”

“*it’s important you get to know the coach as well as you can on an individual work level and how they like information*”

This correlated with the results from Rhind and Jowett (2010) and was further underpinned by the “Openness” facet of Stafford and Canary’s (1991) work.

#### Motivation

Unlike the findings of Rhind and Jowett (2010), Motivation was not the most frequently discussed form of relationship maintenance, although it was mentioned by 100% of participants. It provided 14.4% of the total units and was catergorised into effort, fun, motivate the other and show ability in line with previous literature (Rhind and Jowett, 2010). Effort suggested a commitment to the coach and was highlighted by the following quotes:

“*So results affect you more and you’re more willing to go the extra mile. For example, we lost a game earlier in the season and I was up until*… *I stayed up until. I think it was like 5 o clock in the morning after that night game, changing this match report because I was motivated to help and make things better.*”

“*I think sometimes the best relationships, you probably are willing to do the extra that you might not if you didn’t have that relationship with the coach*”

Motivate the other resulted in quotes that alluded to togetherness of the multidisciplinary team as well as the specific dyad in question. A2 and A4 spoke enthusiastically about this subject:

“*He knows it’s not just always all about him. And that there’s he has a team of staff and he wants to keep them happy, he wants you to want to work for him*”

“*empowered straight away if someone is going to be willing to listen to your opinions based on your analysis that you’ve done. Umm, so yeah, very empowered, very valued from that interaction*”

The following quotes regarding the requirement of the analyst to work hard in order to show ability was mirrored by 4 out of the 6 analysts interviewed.

“*if you keep performing as they asked or above, then they get more faith in you, they’ll give you more respect, more pressure, and then you’ll build a better relationship*”

“*standards remain really high, so the quality of the work you’re producing is still of the standard that’s required that I think that helps maintain that relationship along the way*”

Akin to the findings of Huggan et al. (2015), the analyst’s willingness to provide extra effort based upon the perceived strength of the relationship was also demonstrated. Their findings also allied to the suggestions that portraying motivation also created more supportive colleagues, leading to stronger relationships (Huggan et al., 2015).

#### Preventative Strategies

This dimension was considered important by all participants and provided 16.3% of the total raw units. The sub categories were made up of “Avoiding Conflict,” “Respecting Boundaries” (respecting others role and responsibility within the team) and “Relationship Continuity” (through establishing early understanding and judging dyadic preferences. The relative contribution to these results suggest that the requirement to be separate from Conflict Management was justified (Rhind and Jowett, 2012).

By establishing early understanding of the coaches’ methods, it was perceived that the analyst could adapt their work to suit the preferences of the manager. A4 summarized this by stating:

“*Is to very quickly understand what they require and kind of, shape your work to that because if you don’t, I think that’s when you start to encounter problems*”

3 of 6 interviewees built upon this, suggesting that by having the ability to judge the preferences and character of the coach, they could then ensure the relationship strength was maintained. A3 stressed this by saying:

“*I think you have to get a read on people and rather than chase people round stadiums and pitches with a laptop you need to know when to, when to speak, when you’re allowed to speak, and when to say nothing*”

The concept of respecting boundaries continued the closely associated themes with 5.1% of the data units acknowledging that:

“*Really clear guidelines and like, a real good understanding of what was required from both sides*” (A4)

was key to the success of the relationship. By establishing rules, not only did the relationship reduce the possible conflict scenarios, but also allows for greater motivation from both parties (Jowett and Carpenter, 2015).

Avoiding conflict was subdivided into two themes. “Awareness of Coach Expectations” and “Submissive Avoidance” both showed aspects of the micro-politics associated within this type of dyadic relationship. A2 talked about coach expectations of the analyst:

“*I think delivering, delivering results so like, getting stuff ready or like hitting deadlines etc. – if the coach or manager has asked you for something for a certain time, ya know, have you delivered on time. If you’re failing to deliver then they’ll stop asking you for things, that relationships gunna break down*”

And A3 spoke around submissive avoidance in order to maintain parity within the relationship:

“*So they say, we might do a meeting, and then you know that they won’t, but you have do it anyway, just in case you do have to do it*”

These outcomes add to the current literature surrounding the requirement for the performance analyst to be subordinate to the coach in order for relationships to be maintained (Bampouras et al., 2012; Huggan et al., 2015).

#### Assurance

Assurance was an adaptation from the original COMPASS Model (Rhind and Jowett, 2010) after it replaced “Advice” but it was responsible for the lowest collection of data units, registering just 6% of the total findings. It was however, supported by all participants of the study and primarily revolved around sport communication (12 of 13 total units). Similarly to Preventative Strategies, an emphasis was placed upon the building phase of the relationships to cement the status of the relationship, but data units suggested that they would provide assurances of how they could impact on the success of the athletes rather than being submissive. A6 and A1 supposed:

“*I think probably try and at the very start, in terms of getting an understanding of what they want, and try just to say look this is, this is how I think this could be better*”

“*once that relationship starts building up they will start asking you more and more questions regarding more tactical side of things. So once I had built that relationship up, they were asking me, and I felt open enough to suggest ideas of how meetings could be led and how we can engage players more and how we can get players to get more out of the meetings*”

It was suggested that providing analytical expertise would have a positive effect on the relationship with the coach. A3 and A4 recalled:

“*And he’ll walk in the next day and you have the successful and unsuccessful crosses broken down and you’ll say, ya know, just based on what you said to the other coaches, here’s some bits and pieces and he might show that back to the lads*”

“*quite a lot of our discussion around the pre match was normally verbal, rather than documents if you like so, we would sit an brief the manager on kinda, how we were going to play or how the opposition play*”

There was consensus with previous findings that show how the analyst’s skill-set and alternative viewpoint allowed for greater opportunities for athletes to improve (Hughes, 2004; Francis and Jones, 2014; Huggan et al., 2015).

#### Support

Three of the analysts referenced the personal support that they felt was important within the coach–athlete dyad. A4 provided 3 of the 4 units for this subcategory and suggested:

“*I think respect, kind of a mutual respect between the two is very important*”

This was contradicted, primary by A1 and A2 who believed they were required to give their unconditional support to the coach, as in A2’s words:

“*the gaffer is the one at the top, with the decision making. He’s got the final say on everything, but he’s got to have his own staff, and he’s got to have staff that want to work for him*”

This challenges the findings of Jowett and Cockerill (2003), who perceived that interpersonal relationships were ineffective if the coaches support and knowledge were deemed inadequate by the athlete.

Sport Support was supported with 29 data units (13.5% of the total recorded) from 6 out of 6 analysts’. The majority (24) of these focused around understanding the coaches’ requirements. A3 suggested that part of the responsibility lay with the analyst when they said:

“*you should almost be able to anticipate what the coach wants*”

Whereas A6 had experienced coaches that prescribed the support they required explicitly.

“*I’ve worked with coaches that, they know exactly what they want, and they don’t really want to deviate from that so it’s a case of this is what you’re going to do, this is how you’re going to do it, that’s it!*”

These conflicting viewpoints of the participant have been shown to be important for maintaining relationships as the coach and analyst may need to show leading roles in addition to support roles in certain circumstances throughout the lifespan of the relationship (Jowett et al., 2012a; Yang and Jowett, 2013).

#### Social Networks

Socialization (the social interaction which only included the two members of the dyad) was only alluded to by A1. The 4 (1.9%) examples they provided related to maintaining previous relationships with coaches they are not currently working with:

“*13 of my 14 managers I get on with, still got my back, pick up the phone to them now and speak to them now*”

“*But in a positive way, I’ve been asked to work for previous managers on a few occasions and I’ve had to decline. But I think that shows they value me, and my honesty and my relationship with them*”

This was in stark distinction to previous research which found that 21 of 25 data units were focused upon socializing solely with the coach (Rhind and Jowett, 2010). The participants were mainly sub elite and individual sport focused so may not have been involved within such large sporting environments that employed numerous technical and coaching staff. By showing that maintaining relationship with coaches they were not currently working with professionally, the analyst was able to provide future opportunities for employment within a dyad they perceived as strong and could therefore lead to greater job satisfaction and motivation (Butterworth and Turner, 2014; Huggan et al., 2015).

The remaining units (27–12.6%) were focused around shared networks and were cited by all six analysts. This sub category was further divided into three themes of “Casual Interactions” (non-sport or work-related activities), “Obligatory Socialization” (situations where attendance was mandatory such as end of season awards, or team meals during away travel) and “Socialization Through Sport” (both within work and outside work). A4 defined their interactions with the coach as compulsory:

“*There was always a work kind of emphasis on those kind of*… *You know, if it was a meal or a get together, it was always kind of work related*”

This was in stark contrast to 5 of 6 analysts, including A2, who provided positive examples of casual interactions with the coach:

“*it just gives you that sort of extra, you get to know more about the person, it’s not just always a working relationship. You get to learn a bit more outside which then helps your working relationship*”

Socialization through sport, especially within the confines of the working environment was discussed optimistically on 10 occasions by 4 of the analysts. A6 talked about head tennis matches with staff:

“*the court opens up and it’s a good interaction for staff from different departments, not just coach and analyst but it gets you the opportunity to kind of have a bit of, a bit of fun, a bit of a laughter, a bit of joking around, a bit of competition and you kind of see how the coach reacts in different ways*”

This correlates with findings that social interaction could have positive correlations on the dyad in addition to wider relationships (Jowett and Chaundy, 2004; Rhind and Jowett, 2010). Huggan et al. (2015) also concluded that the social interactions created greater co-operation within the coach–analyst relationship.

### Power Relationships in the Coach–Analyst Relationship

The inductive content analysis provided 142 raw data units related to power relationships and micro-politics (Table 2). Whilst there was data units that aligned to previous research (French and Raven, 1959), especially coercive power:

**TABLE 2:** Summary of results of Power Relationships in the Coach–Analyst Relationship.

**Theme**	**Hierarchical**	**Task focused**	**Social**	**Total**
**Authority**	**37**	**53**	**5**	**95**
%	26.1	37.3	3.5	66.9
No of analysts	5/6	6/6	4/6	6/6
**Equality**	**11**	**19**	**17**	**47**
%	7.7	13.4	12.0	33.1
No of analysts	5/6	6/6	4/6	6/6
**Total**	**48**	**72**	**22**	**142**
%	33.8	50.7	15.5	
No of analysts	6/6	6/6	5/6

“the managers great but ya know, he still the manager and he’s still the one that’s gunna sack ya at the end of the day” (A3)

“the managers more inclined to get rid of you because you don’t have a connection and relationship with him” (A1)

And legitimate power:

“whatever he says in fine. Don’t feel as if you can say, ya know, why don’t we try this instead? I listen to whatever he says, and whether I like it not, you go away and do it which I guess is the point of the manager, but at the same time, my expertise is in analysis. That’s where my expertise lies and I might know more than them, I might know better way to do things but I don’t feel as though I can bring that up because I don’t feel as though they’re going to want to hear it or they’ll shoot me down” (A2)

“I said about just listening and producing exactly what they want rather than what you want to do” (A1)

It was felt the data suggested a very specific type of relationships that was governed by three main higher order themes. These original themes were identified as Hierarchical (thoughts and feelings, non-specific), Task Focused (work related tasks and activities) and Social (non-work related). 66.9% of the points allied to an authoritarian approach whereby the coach was controlling of the relationship. All six analysts provided responses which represented both an authoritarian (Coach dictates) and an equal (where responsibility or behaviors are on a matched level). This was in connection with prior empirical evidence around compliance and co-orientation (Locke, 1985; Purdy et al., 2008).

#### Hierarchical Theme

The following two quotes denoted how the behavior of analyst was dictated by the coach:

“*Be respectful, that’s difficult sometimes when you’re feeling down and your struggling for motivation. At the end of the day, he’s the manager of the football club. Whether he deserves respect or not, you have to give it him because that the nature of the industry we’re in*” (A1)

“*I think a lot of the time, the coach analyst relationship, if one of you is wrong, the analyst has to take it and go ok, well I guess I’m wrong*” (A5)

This form of compliance was responsible for 26.1% of the inductive results and was in-keeping with the findings of Cushion and Jones (2006) and Purdy and Jones (2011). The analysts perceived themselves to be in a similar predicament to the athletes, in that any rebellion against the coaches, may jeopardize their future career within elite sport. In contradiction to these findings, a desire for a more equal relationship was alluded to by just 7.7% of the units suggesting that the coach does command power over the analysts within the workplace.

#### Task Focused Theme

The greatest number of comments were recorded in the Task Focused Authority group and offered 37.3% of the total units analyzed. Comments were recorded from all 6 analyst’s and suggested that most work processes and analysis that was undertaken was controlled by the coach. A2 summarized this section by stating:

“*I listen to whatever he says, and whether I like it not, you go away and do it which I guess is the point of the manager, but at the same time, my expertise is in analysis. That’s where my expertise lies and I might know more than them, I might know better way to do things but I don’t feel as though I can bring that up because I don’t feel as though they’re going to want to hear it or they’ll shoot me down*”

Although sometimes begrudgingly, the analysts showed that they entered into a consented coach dominated scenario which led to a legitimate power relationship (Cushion and Jones, 2006). This category helps to unearth some of the intricacies’ of how the analyst is deployed by coaches within professional football which was considered to be previously sparse (Fernandez-Echeverria et al., 2017).

In repost, A4 provided examples of equality with one particular coach which was perceived by visiting coaches as usual. They suggested that under this coach:

“*it would be very much open and for example, developing a game plan for the weekend. He might say, Oh, I want to play this formation, I would say well, if we play that formation, this might, these are the problems we might encounter. You know, have you thought about doing this?*”

Giddens (1984) argued that within any structure, the persons within must sanction power being exerted over them for that power to be successful. Therefore, if both parties have an alternative mindset, this may lead to a different relationship that deserves further scrutiny.

#### Social Theme

The social theme was the only category which provided greater responses surrounding equality (12%) than authoritative behaviors (3.5%). Just five comments were recorded, which centered around financial authority and humor. A5 said:

“*have a good sense of humor between you, you’re more than happy to take the stick, more than you’re probably giving it, I think it’s quite important*”

Purdy et al. (2008) found that the use of humor and banter between coaches and athletes was often used to instill a belief of influence over the other. They also found that in accordance with Giddens (1984), social interactions were used to change the balance of power and this was validated by the current study with comments being expressed to support this argument:

“*they’re great exercise because the manager stops being the manager and becomes the person that you’re sat in the pub with*” (A3).

## Conclusion

This study outlines that the COMPASS Model, developed through research into the coach–athlete relationship, is transferable across dyads within sport with only minor alterations based upon the roles and responsibilities of analysts. The strategies suggested for maintaining the relationship are centered around the analyst understanding the requirements of the coach to support their needs. By employing a hard working ethos, underpinned by honesty and being approachable, the analysts perceive that the relationship can be productive. The results also highlight the need to employ strategies to prevent conflict occurring as it can lead to the breakdown of relationships if not dealt with swiftly and utilizing the aforementioned behaviors. The respondents also found that socialization through sport and casual interactions was a positive contributor of healthy relationships as it balances the *status quo* of the relationship. This was highlighted by an inductive analysis has also that showcased the existence of authoritative coach behavior’s that, whilst not always appreciated by the analyst, often serve to provide the rules that govern relationships.

The results offer aspiring performance analysts insight into the working relationships within first team elite football environments. This is often perceived as an inaccessible environment due to the secretive nature of professional football and as such, it offers relatively unreported insight. This provides clarity for behaviors which may shape the success and ability of analysts to maintain close working relationships that have been shown to correlate to increased technical and tactical insights for opportunities to improve performance outputs. This paper also provides coaches with knowledge of the perceptions of the performance analyst within this working relationship and how their actions may have a positive or negative effect on the analysts view of the dyad. This may enable coaches to consider their behavior to maintain or enhance their relationships with the analyst and other sport science support staff.

Although the underlying research that developed the COMPASS Model acknowledged the transferability of the conceptualization, only interviewing one half of the investigated dyad may have had an impact on the generalizability of the results. Future studies should look to interview both the coach and analyst to better understand the perspectives and meta perspectives of both persons as the coach would likely have a different portrayal of events. Whilst the use of telephone interviews was justified due to geographical locations of the participants, the inability to view the non-verbal cues of the analyst’s may have caused the researcher to impact upon the flow of conversation.

As this study has investigated a previously unreported area, it should serve as a basis for future research and not be considered conclusive on the subject. Further studies may wish to consider if similar results are found in academy football relationships or across different sports. The identification of power within the relationship may serve as a foundation for future exploration of this phenomena within other relationships within coaches and technical staff within elite sport so that we may provide academia with insight from the applied world.

## Data Availability

The raw data supporting the conclusions of this manuscript will be made available by the authors, without undue reservation, to any qualified researcher.

## Ethics Statement

This study was carried out in accordance with the recommendations of the University of Worcester with written informed consent from all subjects. All subjects gave written informed consent in accordance with the Declaration of Helsinki. The protocol was approved by the University of Worcester.

## Author Contributions

MB organized the data collection and analysis, and provided the draft one. GJ read and edited necessary revisions to the manuscript. Both the authors contributed to the conception and design of the work, and confirmed the version was ready for submission.

## Conflict of Interest Statement

The authors declare that the research was conducted in the absence of any commercial or financial relationships that could be construed as a potential conflict of interest.
