# Supplementary material for: Strategies for Maintaining the Coach–Analyst Relationship Within Professional Football Utilizing the COMPASS Model: The Performance Analyst’s Perspective
Source: Front Psychol. 2019 Sep 10;10:2064. doi: 10.3389/fpsyg.2019.02064 (PMC6748348; doi:10.3389/fpsyg.2019.02064)
Supplement: Supplementary file 1 [file Data_Sheet_1.PDF]

Interview Schedule

What situations can you recall, both in work situations and / or outside of work, when you have engaged in social activities that have included the coach?

(PROBE) What impact does/did that have on your relationship with the coach?

Does/did your coach-analyst relationship allow for open conversations about work processes & the analysis you undertake?

(PROBE) How does/did this affect your motivation in the role?

(PROBE) How does/did this impact on your relationship with the coach

(PROBE) Are you able to talk to your coach about this relationship?

Can you give any examples of when your coach-analyst relationship was not going well?

(PROBE) Was there any actions taken to resolve the situation?

(PROBE) What lessons did you take from this situation?

Can you give any examples of when you felt confident to speak to your coach about something or someone, except them, you were unhappy with?

(YES) Did they offer you any advice or solutions of how to overcome this matter?

(YES)How did this impact your relationship with the coach?

(NO) Why did you feel that you didn’t want to confide in them?
